# Radiomic MRI signature reveals three distinct subtypes of glioblastoma with different clinical and molecular characteristics, offering prognostic value beyond *IDH1*

**DOI:** 10.1038/s41598-018-22739-2

**Published:** 2018-03-23

**Authors:** Saima Rathore, Hamed Akbari, Martin Rozycki, Kalil G. Abdullah, MacLean P. Nasrallah, Zev A. Binder, Ramana V. Davuluri, Robert A. Lustig, Nadia Dahmane, Michel Bilello, Donald M. O’Rourke, Christos Davatzikos

**Affiliations:** 1 Department of Radiology, Perelman School of Medicine, University of Pennsylvania, Philadelphia, PA USA; 2 Center for Biomedical Image Computing and Analytics, Perelman School of Medicine, University of Pennsylvania, Philadelphia, PA USA; 3 Department of Neurosurgery, Perelman School of Medicine, University of Pennsylvania, Philadelphia, PA USA; 4 Department of Pathology and Laboratory Medicine, Perelman School of Medicine, University of Pennsylvania, Philadelphia, PA USA; 5 Department of Biomedical Informatics, Northwestern University Feinberg School of Medicine, Chicago, IL USA; 6 Department of Radiation Oncology, Perelman School of Medicine, University of Pennsylvania, Philadelphia, PA USA

## Abstract

The remarkable heterogeneity of glioblastoma, across patients and over time, is one of the main challenges in precision diagnostics and treatment planning. Non-invasive *in vivo* characterization of this heterogeneity using imaging could assist in understanding disease subtypes, as well as in risk-stratification and treatment planning of glioblastoma. The current study leveraged advanced imaging analytics and radiomic approaches applied to multi-parametric MRI of *de novo* glioblastoma patients (*n* = 208 discovery, *n* = 53 replication), and discovered three distinct and reproducible imaging subtypes of glioblastoma, with differential clinical outcome and underlying molecular characteristics, including isocitrate dehydrogenase-1 (*IDH1*), O^6^-methylguanine–DNA methyltransferase, epidermal growth factor receptor variant III (*EGFRvIII*), and transcriptomic subtype composition. The subtypes provided risk-stratification substantially beyond that provided by WHO classifications. Within *IDH1*-wildtype tumors, our subtypes revealed different survival (*p* < 0.001), thereby highlighting the synergistic consideration of molecular and imaging measures for prognostication. Moreover, the imaging characteristics suggest that subtype-specific treatment of peritumoral infiltrated brain tissue might be more effective than current uniform standard-of-care. Finally, our analysis found subtype-specific radiogenomic signatures of *EGFRvIII*-mutated tumors. The identified subtypes and their clinical and molecular correlates provide an *in vivo* portrait of phenotypic heterogeneity in glioblastoma, which points to the need for precision diagnostics and personalized treatment.

## Introduction

Precision diagnostics, prognostication, and personalized treatment in cancer patients call for finer characterization of tumors than current practice. Multi-parametric magnetic resonance imaging (mpMRI) is a powerful diagnostic tool that can facilitate *in vivo* characterization of diverse aspects of the tumor and its micro-environment^1,2^. In this study, we aimed to characterize the heterogeneity of glioblastoma, which is the most aggressive adult primary brain tumor with a reported median survival of only about 14 months^3^. Glioblastoma exhibits significant molecular, histological, and imaging heterogeneity across and within patients, as well as variable proliferation, which poses several diagnostic and therapeutic challenges^4,5^. Current standard of care^6^ is generally uniform across glioblastoma patients and does not take into account the patient specific characteristics. In fact, the heterogeneous landscape, different response to the same treatment^7^, and resistance to standard treatment regimens render the “same treatment for all” approach inadequate. Thus, accurate non-invasive characterization of the heterogeneity of glioblastoma is critical not only for better understanding of this poor-prognosis cancer, but also for developing personalized therapies to improve patient outcome, and for facilitating targeted enrollment into clinical trials.

Past efforts have documented evidences of the heterogeneity among glioblastoma patients captured within peritumoral edema using conventional MRIs^8^, followed by using additional features from other tumor regions^1,2,9^. However, as tumors are spatially and temporally heterogeneous, extracting comprehensive measures of the entire tumor and peritumoral tissue using mpMRI is likely to provide better characterization of patients, which might not be captured by employing conventional modalities or global measures.

The goal of this study is to systematically investigate imaging heterogeneity in patients with *de novo* glioblastoma, by radiomic analysis of pre-operative mpMRI data. We conducted this analysis by applying a high-dimensional clustering on a comprehensive set of features, reflecting imaging surrogates of tumor progression, angiogenesis, proliferation, cellularity, and peritumoral infiltration. Specifically, we hypothesized that pattern analysis methods applied to mpMRI would be able to identify complex and otherwise visually difficult to appreciate imaging subtypes of glioblastoma that relate to prognosis and underlying molecular characteristics of the tumor.

The current work differs from prior studies employing machine learning tools to derive specific predictions^10–13^, in that it employs completely data-driven analyses of very extensive imaging mpMRI feature sets to dissect phenotypic heterogeneity, without any a priori knowledge or target of molecular and clinical characteristics. Understanding these subtypes may provide a mean to elucidate underlying heterogeneity in their molecular composition and microenvironment, and may lead to personalized treatment planning.

## Results

### Reproducible clustering points to three distinct imaging subtypes

An extensive set of 267 features was extracted from enhancing tumor (TU), non-enhancing core (NC), and edema (ED). The features for the three tumor subregions were calculated by using conventional imaging modalities such as T1-weighted (T1), T2-weighetd (T2), T1 with contrast-enhanced (T1CE), and T2 fluid-attenuated inversion recovery (FLAIR), dynamic susceptibility contrast-enhanced (DSC)-MRI based measures exclude (modalities) such as relative cerebral blood volume (rCBV), percent signal recovery (PSR) and peak height (PH), and diffusion tensor imaging (DTI) based measures such as axial diffusivity (AX), radial diffusivity (RAD), fractional anisotropy (FA) and trace (TR) the measures derived from diffusion and perfusion imaging were used as independent modalities.

**Figure 1:**
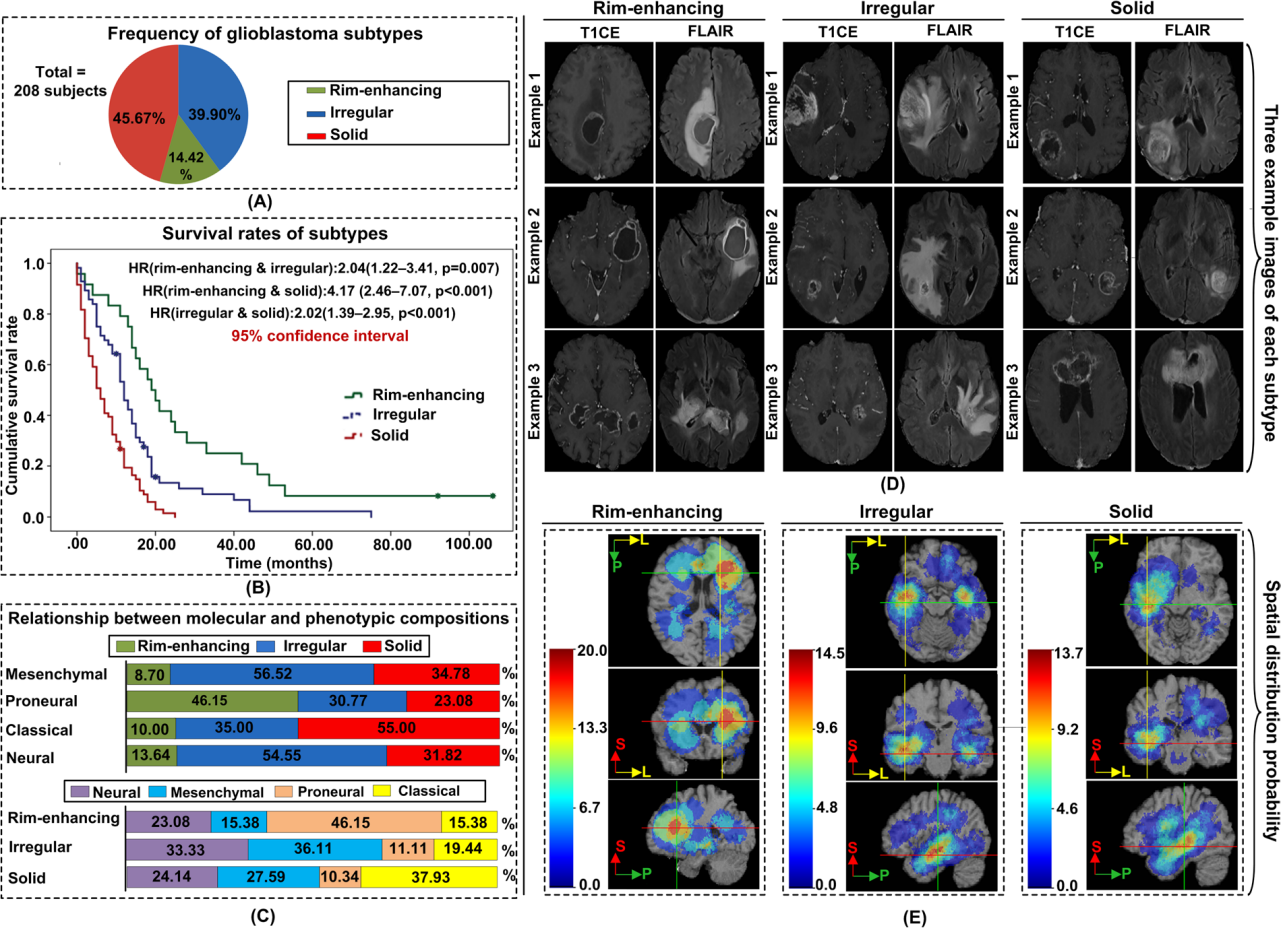
Glioblastoma imaging subtypes identified by the clustering process. (**A**) The frequency of each subtype. (**B**) Kaplan–Meier survival curves of the subtypes. (**C**) Relationship between the molecular composition (Neural/Mesenchymal/Proneural/Classical) and imaging subtypes (rim-enhancing/irregular/solid). (**D**) Three representative subjects of each subtype (closest to the mean of the cluster). (**E**) Spatial distribution probability of the tumors of each subtype. The color look-up tables show the probability of tumor existence. HR = Hazard ratio.

Three distinct and reproducible clusters exhibiting different imaging subtypes of glioblastoma emerged as a result of K-means clustering process, which was repeated 1,000 times to determine robust and reproducible subtypes. We named the three discovered subtypes based on their prominent quantitative imaging characteristics: rim-enhancing, irregular and solid (Fig. 1D). Rim-enhancing subtype was characterized by a hyper-intense rim-enhancing tumor, with lower cell density, medium-sized edema, relatively spherical shape, and less neovascularization, compared to other subtypes. Irregular subtype had irregular edges, moderate cell density and neovascularization, infiltrated peritumoral edematous tissue that was large and irregularly shaped, and which had MR signals indicating high fluid concentration, suggesting deep but less dense infiltration. Solid subtype was highly uniformly vascularized, had the highest cell densities, small-sized edema, was moderately spherical and well-circumscribed with peritumoral edematous tissue that showed signs of heterogeneous neovascularization, compared to other subtypes.

The average adjusted rand index, which measures cluster reproducibility, for K varying from 2 to 6 was 0.78, 0.84, 0.66, 0.63 and 0.59, respectively. The 10-fold cross-validation of the discovered clusters led to 88% clustering assignment reproducibility. The percentage distribution of 208 subjects in rim-enhancing, irregular, and solid clusters, respectively, was 14.42%, 39.90% and 45.67%.

### Survival analysis of subtypes

The solid subtype was associated with the worst survival (median survival (MS) = 6 months) while the irregular subtype had an intermediate survival (MS = 12 months) and the rim-enhancing subtype had the longest survival (MS = 19 months). Kaplan-Meier curves (Fig. 1B) demonstrated significant differences in the survival rates of different subtypes (*p* < 0.001 using Log-Rank (Mantel-Cox)). The hazard ratio (HR) between rim-enhancing and solid subtypes was 4.174 (2.463–7.072, *p* < 0.001); rim-enhancing and irregular subtypes was 2.037 (1.219–3.405, *p* = 0.007); and irregular and solid subtypes was 2.021 (1.388–2.945, *p* < 0.001). These survival estimates were better than the existing survival estimates, based on molecular measures^14^. Fig. S4 shows Kaplan-Meier curves for the molecular subtypes and the corresponding HR.

### Important phenotypic characteristics

**Figure 2:**
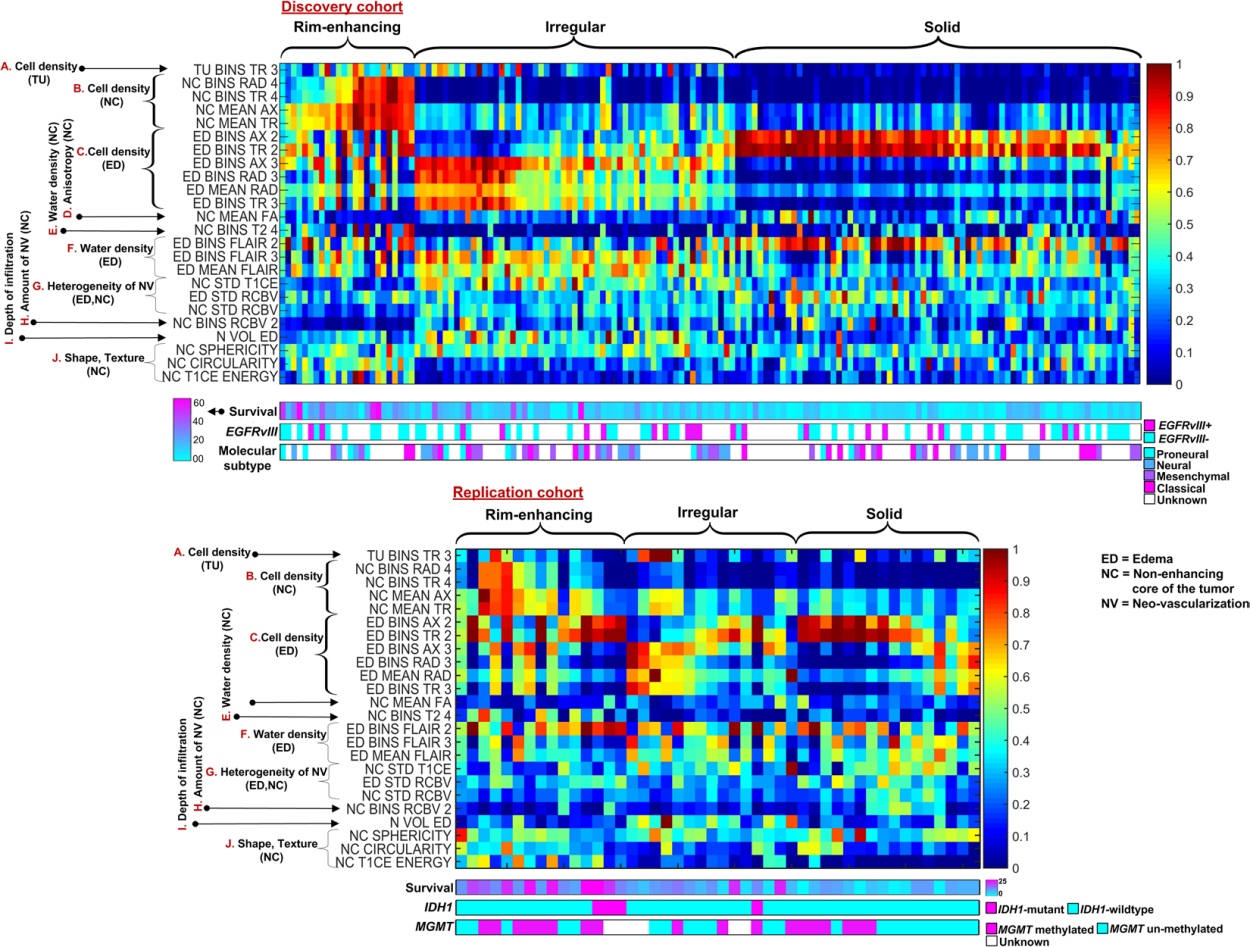
Identification of intrinsic imaging subtypes of glioblastoma using unsupervised clustering. Upper half of the figure shows heat map of the discovery cohort with columns representing subtypes (subjects) and rows representing features. Underneath the heat map are the color-coded survival rates, epidermal growth factor recipient variant -III (*EGFRvIII*) mutation status and molecular subtype for these subjects. The lower half of the figure shows heat map of the replication cohort with columns representing subtypes (subject) and rows representing features. Underneath the heat map are the color-coded values of survival rates, *IDH1* mutation status and *MGMT* methylation status. The survival rates after 98th percentile were replaced with that value to alleviate the effect of outliers (long survivors) in the color bar. Only the subjects having gross total resection were shown here.

The analysis of image features significantly associated with each glioblastoma imaging subtype revealed representation of all the modalities (Fig. 2, Fig. S1). Most of these features were extracted from the intensity histograms. For instance, ‘TumorSubregion_BINS_Modality_BinNumber’ is the percentage of voxels in the ‘*BinNumber*’ of the intensity distribution of a certain ‘Modality’ for a certain ‘TumorSubregion’. Similarly, ‘TumorSubregion*_*MEAN_Modality’ and ‘TumorSubregion_STD_Modality’, respectively, are the mean and standard deviation of intensity in a certain ‘Modality’ for a certain ‘TumorSubregion’ (Table S1). The first set of features summarized statistics from measures derived from DTI, which reflected higher TR in the enhancing and non-enhancing tumor core (Fig. 2A,B, Fig. S1A,B) and lower FA in non-enhancing tumor core of rim-enhancing subtype (Fig. 2D, Fig. S1D), and lower TR in the peritumoral edema of solid subtype (Fig. 2C, Fig. S1C). The second set of features comprised measures from structural MRI. These measures related to water density (Fig. 2E,F, Fig. S1E,F), where relatively higher water density was observed in the edema of irregular subtype, and to infiltration (Fig. 2I, Fig. S1I) and appearance of tumor such as volume, shape, irregularity, and texture (Fig. 2J, Fig. S1J). The rim-enhancing subtype included more homogeneously textured and regularly shaped tumors compared to other subtypes. The third category of features summarized characteristics calculated from DSC-MRI signal, and showed relatively homogeneous and lower rCBV in rim-enhancing subtype compared to others (Fig. 2G,H, Fig. S1G,H).

The most distinctive features, shown in Fig. 2, reflect representation of all the modalities. This observation underscores the advantage of using a mpMRI model that integrates synergistic imaging features extracted from various imaging modalities. The main imaging, molecular and prognostic characteristics of these subtypes are summarized in Table 1.

**Table 1:** Imaging, prognostic, and molecular characteristics of the three imaging subtypes of glioblastoma.

	Rim-enhancing	Irregular	Solid
*Discovery and replication cohorts*			
Imaging characteristics	Lower cell density	Moderate cell density	High cell density, dense peritumoral infiltration
	Lower angiogenesis	Moderate angiogenesis	High angiogenesis
	Lower micro-vascularity	Moderate micro-vascularity	High micro-vascularity
	Medium-size edema	Large-size and irregular edema (more infiltration)	Small-size edema
	Highly spherical	Least spherical (Highly irregular edges)	Moderately spherical, well circumscribed
*Discovery cohort*			
Prognosis (median overall survival in months)	19	12	6
Distribution in overall population (%)	14.42	39.90	45.67
Predominant molecular subtype in this imaging subtype	Proneural	Neural and Mesenchymal	Classical
Localization	Frontal lobe	Left and right perisylvian temporal lobe	Right perisylvian temporal lobe
*Replication cohort*			
Prognosis (median overall survival in months)	18	11	7.5
Distribution in overall population (%)	32.69	30.76	36.53

### Molecular composition of subtypes

The percentages of classical, mesenchymal, proneural, and neural subtypes in the discovery cohort were 25.64%, 29.49%, 16.67% and 28.21%, respectively. The relationship between the molecular composition and imaging subtypes (Fig. 1C.) demonstrated that the proneural tumors were more prevalent in the rim-enhancing subtype (having best prognosis), while more than 50% of neural and mesenchymal tumors were of the irregular subtype that showed intermediate prognosis. Classical tumors were frequently associated with the solid subtype that showed worst clinical outcome. The distribution of molecular subtypes differed significantly amongst imaging subtypes (*p* < 0.05 using Chi-Square distribution).

### Spatial distribution of tumors for different subtypes

The three subtypes showed marked differences in tumor localization (Fig. 1E). The rim-enhancing tumors had a clear predilection for the frontal lobe, especially on the left side. The solid tumors seemed to have a focused preference for right temporal lobe. The irregular tumors, on the other hand, were found both in the left and right temporal lobes. Parietal, especially peri-ventricular, regions were also somewhat involved in all subtypes.

### Better imaging-based identification of epidermal growth factor receptor variant –III (*EGFRvIII*)-mutated tumors within homogeneous subtypes

The prevalence of *EGFRvIII*-mutated tumors was 12.50%, 39.02%, and 30.61%, respectively, in rim-enhancing, irregular and solid subtypes. To test our hypothesis that imaging-based estimation of tumors with and without *EGFRvIII* is better within subtypes, as opposed to when compared across all the patients, we performed a uni-variate (effect-size) and a multi-variate analysis (support vector machines (SVM)).

The higher values of effect-sizes for irregular and solid subtypes (Fig. S2) indicate that tumors with and without *EGFRvIII* were more distinct within subtypes rather than across all the patients, despite the drastic reduction of sample size within each subtype, compared to the pooled set. It is also notable that the biomarkers that identify tumors with and without *EGFRvIII* (Fig. S2) were very different in the irregular and solid subtypes, with various perfusion measures being critical predictors of the mutation in the former and unimportant in the latter. In the multi-variate analysis carried out using nonlinear SVM, the classification within subtypes was more successful with average accuracy of 80.19% (87.50%, 75.61% and 81.63% for rim-enhancing, irregular and solid subtypes) compared to the classification carried out across all the patients exhibiting an accuracy of 73.58%. Based on the low prevalence of *EGFRvIII*-mutated tumors in rim-enhancing subtype compared to irregular and solid subtypes, we hypothesized that rim-enhancing subtype is a good indicator of lack of *EGFRvIII*. Therefore, we established a null model based on which we assigned every subject of rim-enhancing subtype to ‘tumors without *EGFRvIII*’ category. We did not make any classifier for rim-enhancing subtype to identify tumors with and without *EGFRvIII* mutation.

### Reproducibility of imaging subtypes in replication cohort

**Figure 3:**
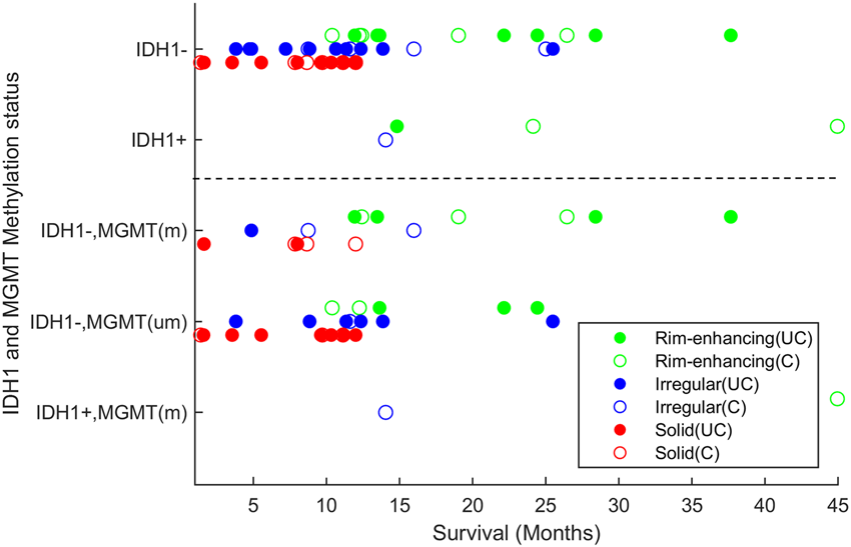
Survival analysis of the replication cohort. The identifiers (m) and (um) on the vertical axis, respectively, show *MGMT* methylated and un-methylated subjects. The identifiers C and UC in the legend entries, respectively, show censored (which were all alive at the time of last recorded follow-up or record check) and uncensored subjects (death was confirmed). *IDH1*+ and *IDH1*−, respectively, show *IDH1*-mutated and *IDH1*-wildtype patients.

The distribution of replication cohort in rim-enhancing, irregular and solid subtypes, respectively, was 32.69%, 30.76% and 36.53%. The median survival for the rim-enhancing, irregular and solid subtypes was 18, 11, and 7.5 months, respectively (Fig. S5). The three subtypes differed significantly in terms of survival rates (*p* < 0.001 using Log-Rank (Mantel-Cox)). The HR between rim-enhancing and solid subtypes was 10.62 (3.34–33.81, *p* < 0.001); rim-enhancing and irregular subtypes was 3.22 (1.15–9.08, *p* = 0.02); and irregular and solid subtypes was 3.29 (1.27–8.49, *p* = 0.014). A summary of survival rates, isocitrate dehydrogenase-1 (*IDH1*) mutation and O^6^-methylguanine–DNA methyltransferase (*MGMT*) status of the replication cohort is given in Fig. 3 and the detailed results are given in Table 2.

**Table 2:** Median survival (in months), categorized by *IDH1* mutantation expression and *MGMT* methylation status for the subjects of the three imaging subtypes, separately for the censored (survival was at least as high, as these patients were alive at last time-point on record) and uncensored (exact survival) cases of replication cohort.

Imaging subtypes	*IDH1* status (n)		*MGMT* status (n)			Total
	*IDH1-*	*IDH1-*	Methylated	Un-methylated	Unknown	
	mutant	wildtype				
**Rim-enhancing**						
Uncensored	14 (1)	21 (7)	20 (4)	21 (3)	14 (1)	17.5 (8)
Censored	33.5 (2)	12 (5)	22 (4)	11 (2)	23 (1)	18 (7)
Total	23 (3)	15.5 (12)	22 (8)	13 (5)	18.5 (2)	18 (15)
**Irregular**						
Uncensored	—	9 (10)	4 (1)	11.5 (6)	7 (3)	9 (10)
Censored	13 (1)	13 (4)	13 (3)	11 (1)	24 (1)	13 (5)
Total	13 (1)	10.5 (14)	10.5 (4)	11 (7)	8.5 (4)	11 (15)
**Solid**						
Uncensored	—	7 (9)	4 (2)	9 (7)	—	7 (9)
Censored	—	8 (7)	7.5 (4)	9 (3)	—	8 (7)
Total	—	7.5 (16)	7 (6)	9 (10)	—	7.5 (16)

Current WHO classifications of glioblastoma include *IDH* mutation status, due to its association with better prognosis. WHO considers all the *IDH1*-wildtype patients, which are roughly 95% of the glioblastoma patient population, to have relatively poor prognosis. However, our method shows a remarkable heterogeneity within *IDH1*-wildtype patients as we observe three distinct sets of patients: rim-enhancing, irregular and solid (Fig. 3, top row). This observation highlights the complementary value of imaging to the established WHO classification for patients’ prognosis. The Kaplan-Meier curves (Fig.S6) also reveal three distinct subtypes within *IDH1*-wildtype patients (*p* < 0.001, log-rank test; HR = 3.38, 95% CI: 2.64–4.34).

### Subtypes reveal complementary information on survival prediction

The Cox proportional hazard models developed on age (1 features), location (9 features), and imaging subtype (1 feature) individually were compared with the integrative model in which imaging subtype was combined with the others. The concordance index (c-index), respectively, was 0.671 (*p* < 0.01), 0.608 (*p* = 0.03) and 0.646 (*p* < 0.01) for age, location and imaging subtypes, whereas jointly considering subtype, age or location improved the c-index to 0.717 (*p* < 0.01) and 0.715 (*p* < 0.01), respectively. A model including imaging subtypes, age and location all had the highest c-index of 0.741 (*p* < 0.01). Similarly, the combination of subtype with *IDH1* mutation status in the replication cohort also improved c-index by 0.752 (*p* < 0.01) compared to 0.559 (*p* = 0.08) and 0.737 (*p* < 0.01), respectively, provided by *IDH1* mutation status and subtype alone. The better survival prediction by combining *IDH1* mutation status and subtype information highlight the complementary value of imaging to the established WHO classification for patients’ prognosis, albeit subtype was markedly the best predictor when used individually.

## Discussion

In this study, we used advanced multivariate imaging analysis methods in a large cohort of glioblastoma patients having undergone mpMRI, and found remarkable phenotypic heterogeneity of this cancer, captured by an extensive set of radiomic features. Moreover, we identified three distinct and reproducible imaging subtypes showing differential characteristics in terms of overall survival rates, anatomical location, molecular composition, and radiological measures of cell-density, vascularization, infiltration, and extent of tumor. These results characterize the anatomical and physiological phenotypic heterogeneity of glioblastoma in a systematic way, and suggest that its evaluation should consider this subtype for a patient under consideration.

From a precision diagnostics perspective, our results indicate that sub-categorization of glioblastoma by taking both *IDH1* mutation status and imaging subtype might provide a more precise diagnosis, as well as more accurate prognostication. From a personalized treatment perspective, our results indicate that subtype-specific treatments might be more effective than current standard-of-care approaches. In particular, the solid subtype was found to have a very spatially confined region of peritumoral infiltration, albeit with markedly more tumor-like characteristics, compared to the irregular subtype, suggesting a likely rapid transition to solid tumor. This subtype might therefore benefit from very aggressive peritumoral resection and radiation dose escalation, especially in view of its poor survival. The irregular subtype, on the other hand, displayed a much more migratory and deep, but less dense, infiltration phenotype, and may be less likely to benefit from more aggressive peritumoral treatment than that offered by standard-of-care.

### Interpretation of the three imaging subtypes and correlation to prognosis

Considering the distinctive characteristics of the subtypes, we sought to shed light on imaging signatures which are significantly different among subtypes and correlate well to prognosis. The diffusion measures (Fig. 2A–D, Fig. S1A–D) provide information on cell density of the tumor and peritumoral tissue, which is of prognostic value^15^. Tumors with high cellularity tend to have lower TR^16^ and higher FA^15^, and are associated with poor prognosis^17^. Consistent with the existing literature on survival^10,17^, the rim-enhancing subtype, which has larger regions of lower FA and cell density (higher TR) determined by the histograms, has relatively favorable survival, whereas the solid subtype, which shows larger regions of higher FA and cell density (lower TR), has shortest survival. Critically, the peritumoral edematous infiltrated tissue showed very pronounced differences between the solid and the irregular subtypes, with the former displaying relatively denser tissue (Fig. 2C, Fig. S1C). Consistent with similar findings from the FLAIR signal’s water density measures (Fig. 2F, Fig. S1F), and considered together with the fact that solid subtype had the smallest peritumoral edema (Fig. 2I, Fig. S1I), this result would be consistent with the solid subtype being a very aggressively but locally growing tumor, with a densely infiltrated proximal peritumoral region. Conversely, the irregular subtype would be more consistent with a deeply migrating, albeit less densely infiltrating, tumor (Fig. S3).

The neovascularization measures have shown strong associations with clinical outcome of glioblastoma. In an earlier study, rCBV parameters were found to be significantly higher (*p* < 0.05) in older patients (> = 65) and were independently associated with shorter survival compared to younger patients (<65)^18^. Similarly, patients who were predicted to survive longer had relatively lower tissue volumes with increased PH, which relates to increased and compromised microvascularity^10^. In our study, the neovascularization measures also led to some important findings (Fig. 2G,H, Fig. S1G-H). The rCBV and T1CE relate to tumor angiogenesis, contrast agent leakage, size of extravascular space, and rate of blood flow^19,20^. Similar to previous studies^10,18^, the rim-enhancing subtype, which shows larger regions of lower rCBV has favorable survival, whereas the solid subtype, which shows larger regions of higher rCBV, has shortest survival. Critically, the solid subtype displayed characteristics consistent with uniformly and highly vascularized tumor, whereas the irregular subtype was markedly more heterogeneous in its T1CE signal, which was also visually apparent (Fig. 1D, top row). Interestingly, peritumoral edematous tissue showed signs of heterogeneous neovascularization in the solid subtype, also consistent with its increased cell density measures and with a subtype of aggressively, but locally infiltrating tumor.

Morphological aspects of heterogeneity and invasiveness were quantified via appearance related features of the tumor (volume/shape/texture). The rim-enhancing, irregular, and solid subtypes, respectively, demonstrated high, low, and medium sphericity, circularity and texture homogeneity (Fig. 2J, Fig. S1J), and medium-, large-, and small-size edema (Fig. 2I, Fig. S1I), respectively. This implies that the rim-enhancing subtype has regular and homogeneous appearance, whereas the irregular subtype has highly irregular and heterogeneous appearance. Consistent to a previous study^1^, the rim-enhancing subtype, which shows higher regularity and homogeneity, has favorable survival, whereas the irregular subtype, which shows lower regularity and homogeneity, has shorter survival.

The possible explanation of the favorable prognosis of rim-enhancing subtype may be their limited invasion in the peritumoral tissues compared to heterogeneous and irregular-shaped tumors of irregular subtype, which seem to be more infiltrative. This result suggests that appearance of tumor calculated via structural MRIs is not the only factor contributing toward prognosis. Rather, multiple factors including diffusion and perfusion characteristics of the tumor, collectively determine aggressive biologic behavior and poor prognosis, thereby providing support for use of advanced MRI protocols.

### Correlation of imaging subtypes with spatial location and molecular subtype

Increasing attention has been recently paid to the spatial distribution of glioblastomas^21,22^, an aspect that had remained relatively underappreciated previously. The identified subtypes displayed differences in predilection to spatial location; the rim-enhancing subtype that appeared predominantly in frontal lobe has longer survival, which is in accordance with existing literature^23,24^. A partial explanation of the relatively longer survival of these patients is the ability to more aggressively resect frontal regions, albeit the differences in *IDH1* prevalence and molecular subtype composition suggest additional reasons for increased survival of those patients. The solid subtype is more frequent in the temporal lobe, in close proximity to sub-ventricular zone (SVZ). Tumors close to SVZ show favorable growth/infiltration capacities^25^ and decreased survival possibly due to the proximity to stem cells as well as to dense subcortical fibers along which they can migrate^26,27^. The close proximity to SVZ also suggests that these tumors may derive from stem and/or progenitor cells and therefore have cellular and molecular characteristics that lead to increased aggressiveness.

Our results suggest that the molecular heterogeneity of glioblastoma^14^ relates, somewhat, to the imaging heterogeneity captured herein. The major findings in this direction were that the proneural and classical signatures were more prevalent in rim-enhancing and solid subtypes, respectively, which is in agreement with the relatively better prognosis of proneural and worse prognosis of classical tumor^28^. Further, more than 50% of neural and mesenchymal tumors belonged to irregular subtype, consistent with relatively intermediate survival. Although these results indicate some degree of mapping between molecular and imaging subtypes, this mapping is by far not one-to-one, which further underlines the complementary, and potentially synergistic, value of imaging and molecular subtyping.

### Potentially more effective radiogenomic signatures are derived from phenotypically uniform clusters: *EGFRvIII* classification

The *EGFRvIII* classification provided a promising indication that imaging signatures of certain mutations might be better identified within a subtype rather than universally across all patients, albeit only this specific mutation was investigated herein. Our results also suggest that the mechanisms by which *EGFRvIII* mutation alters the tumor’s phenotype might vary across subtypes, potentially reflecting complex interactions among genetic and micro-environmental factors influencing the tumor’s growth/infiltration. In particular, several perfusion-derived features were important markers of *EGFRvIII* mutation in the irregular subtype. These features showed agreement with higher magnitude and spatial heterogeneity of perfusion signals in *EGFRvIII*-mutated tumors and demonstrated increased tumor vascularization. In sharp contrast, important markers of *EGFRvIII*-mutated tumors in the solid subtype were diffusion and FLAIR signals. These features are consistent with relatively decreased cell density and increased water concentration in *EGFRvIII*-mutated tumors, and might reflect the migratory nature of *EGFRvIII*-mutated tumors in relation to imaging discussed in a recent study^29^. Considering that the solid subtype has imaging signals consistent with high cell density in the tumor, this result could potentially suggest that in the solid subtype, *EGFRvIII* mutation might enable higher motility in otherwise tightly packed tumor cells (Fig. S2).

### Synergistic value of imaging and genetic information

Current WHO classifications include *IDH* mutation status, due to its association with better prognosis. Three out of four *IDH1*-mutants in our study belonged to the rim-enhancing subtype, consistent with long survival of rim-enhancing subtype. Interestingly, the *IDH1*-mutated patient with the shortest survival belonged to the irregular subtype, which overall had lower survival, indicating that imaging subtype can potentially add predictive value within *IDH1*-mutated patients. More importantly, however, a clear prognostic benefit is achieved if the *IDH1*-wildtype patients are further refined based on our results (e.g. Fig. 3) into “rim-enhancing” and “other”, due to the marked survival difference between these categories. In fact, *IDH1*-wildtype patients that belong to the rim-enhancing subtype have similar survival to that of *IDH1*-mutated patients. In addition, the results revealed that sole *IDH1* mutation status and imaging subtype, respectively, provided c-index of 0.559 (*p* = 0.08) and 0.737 (*p* < 0.01) for survival prediction, whereas the prediction was improved to 0.752 (*p* < 0.01) after combining both. This underlines the complementary and synergistic, value of imaging and genetic information.

### Clinical value for precision diagnostics

The current study provides a non-invasive and reproducible method for pre-operative characterization of glioblastomas into different subtypes. Even at this early stage of the molecular understanding of glioblastoma, statistically significant correlations are seen between our imaging subtypes and the four molecular subtypes, as well as *EGFRvIII* and *IDH1* mutation status, and *MGMT* methylation status. As the genetic understanding of glioblastoma evolves, the correlations may be refined, leading to improved diagnostic accuracy and potentially improved outcomes.

The use of the standard imaging sequences renders our study likely to be readily translated to clinical workflow and to contribute to precision diagnostics. Further, the generality of the employed methodology makes it suitable for application to other types of cancer. Since imaging captures spatial heterogeneity of the tumor, and is repeatedly used over time to monitor evaluation of glioblastoma in patients both after treatment and with recurrent tumors, the proposed imaging-based approach for stratification can potentially aid in all phases of care of the glioblastoma patients, including the selection of appropriate patient sub-groups into clinical trials and the dynamic adaptation of treatment approach.

### Limitations

A limitation of this study is that the data were acquired from a single institution, whereas multicenter data would be beneficial to further and externally validate our imaging subtypes. However, the use of independent discovery and replication cohorts, along with the use of clinical mpMRI protocols, provides confidence that these subtypes will generalize well to other institutions and patient populations. Another limitation of our study is the unavailability of genetic information such as *IDH1* and *MGMT* mutation status for discovery cohort. In addition, our study lacks voxel-by-voxel histopathologic ground truth from the tumor and infiltrated peritumoral edema region.

## Materials and Methods

### Study setting and data source

All experiments were approved by the Institutional Review Board (IRB) of the University of Pennsylvania (approval no: 706564) and written informed consent was obtained from all patients. All experiments were carried out in accordance with the guidelines and regulations of the approved IRB.

#### Dataset

We first analyzed a discovery cohort of 208 patients with *de novo* glioblastoma, who were diagnosed at the University of Pennsylvania between 2006 and 2013 and having available pre-operative MRI, consisting of T1, T2, T1CE, FLAIR, DSC-MRI, and DTI. A replication cohort of 53 *de novo* glioblastoma patients was also acquired between 2013 and 2016. Details on image acquisition, and inclusion criteria/data source can be found in Section S1 and S2, respectively. The demographics are provided in Table S2 and Table S3, whereas KPS scores are given in Table S4.

#### Pre-processing applied on the dataset

**Figure 4:**
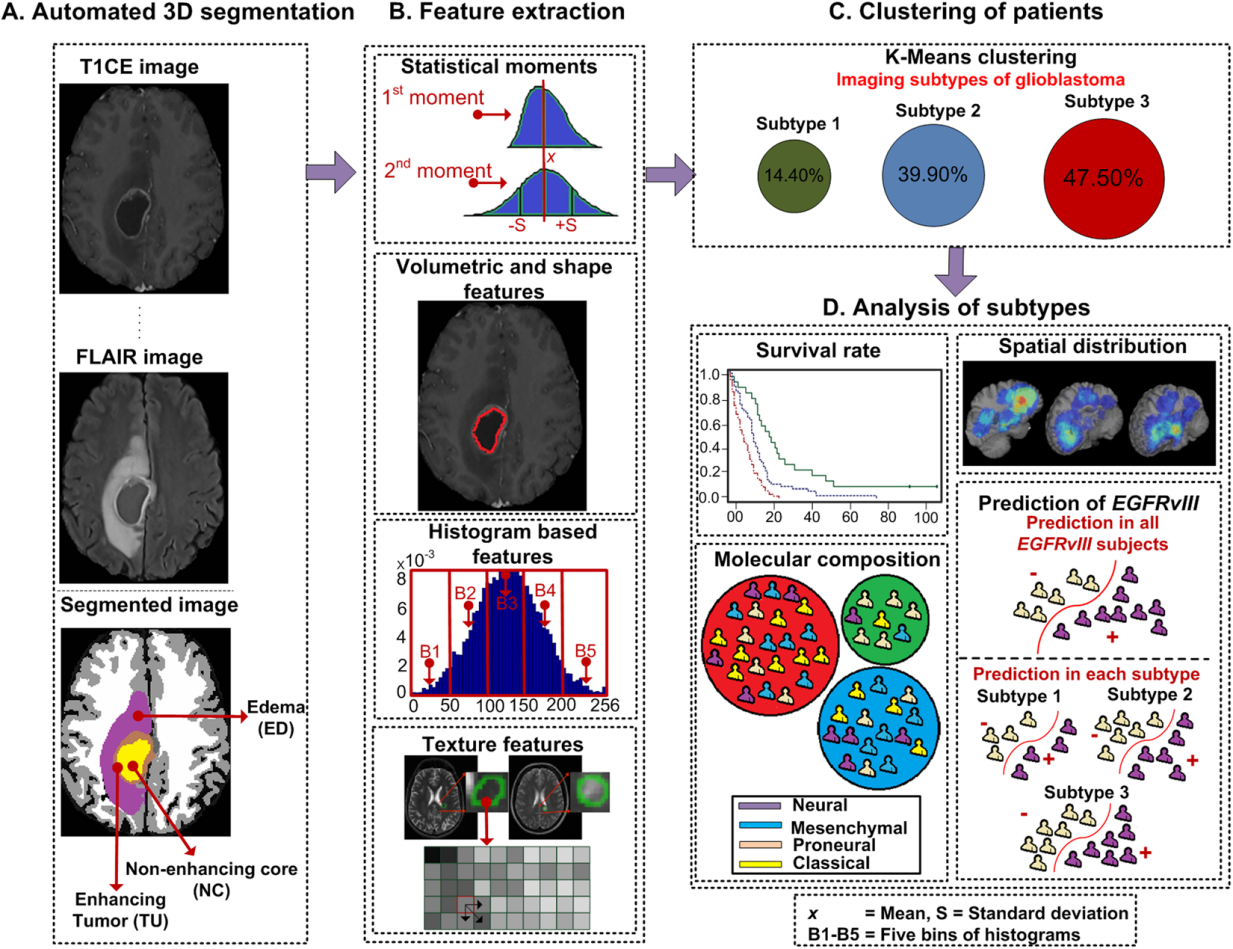
Image post-processing workflow. (**A**) Pre-processed images (examples: T1CE, FLAIR) and segmentations. (**B**) = Extracted radiomic features calculated in all images in the segmented regions (ED, TU, NC). (**C**) K-Means clustering. (**D**) Analysis of the identified imaging subtypes of glioblastoma in terms of overall survival rates, spatial distribution, molecular subtype composition, and *EGFRvIII* prediction.

All MRIs of each patient were co-registered, smoothed, corrected for magnetic field in-homogeneities, and skull stripped^30–32^. The computer-based glioma image segmentation and registration algorithm (GLISTR)^33,34^ was used to segment TU, NC, and ED. The GLISTR segmentations were confirmed by the expert readers and were revised before image analysis, if necessary. GLISTR was also used to estimate parameters of a biophysical glioma growth model^33,35^ and to co-register patient data with a standardized atlas for tumor spatial location quantification. An overall schematic of the proposed method is given in Fig. 4.

### MRI features

For each patient, we extracted multiple features from T1, T1CE, T2, FLAIR, AX, FA, RAD, TR, rCBV, PH, and PSR in order to capture various phenotypic characteristics of TU, NC, and ED. Specifically, the features included: i) volumetric and shape, ii) intensity, iii) histogram, iv) texture, and v) tumor growth parameters.

#### Volumetric and shape features

Volumetric features comprise total brain size, and sizes of various tumor subregions (ET, NC and TU). These features were measured in number of voxels and were normalized with total brain size. In addition, ratios of the sizes of various tumor subregions were also calculated:

- Ratio of the size of ED to whole tumor (ED + NC + TU).
- Ratio of the size of TU to NC.

In addition, circularity (2D) and sphericity (3D) were used to quantify the shape of NC, NC + TU and NC + TU + ED.

#### Intensity features

The intensity features describe the first-order statistical distribution of the voxel intensities within the tumor. They comprise mean and standard deviation of the voxel intensities in tumor subregions (ED, NC, TU) in all the imaging sequences (T1, T1CE, T2, T2-FLAIR, AX, FA, RAD, TR, rCBV, PH, PSR).

#### Histogram features

Histograms, reflect various imaging signal distributions within different delineated tumor subregions. The shapes of these histograms express anatomical and functional changes caused by the tumor that result in signal changes and have demonstrated a connection to clinical endpoints, such as survival, risk factors, and underlying cancer molecular characteristics^10^. Here, we divide the signal distribution of each subregion in each imaging sequence in five distribution bins and calculate the percentage of voxels in each distribution bin.

#### Texture features

The texture features describe the second-order statistical distribution of the voxel intensities within the tumor and were computed from a gray-level co-occurrence matrix (GLCM)^36^. To obtain these features, the image volumes were firstly normalized to 32 different gray levels, and then a bounding box of 5 × 5 × 5 voxels was used for all the voxels of each image as a sliding window. Then, a GLCM was populated by taking into account the intensity values within a radius of 2 pixels and for the 26 main 3D directions to extract texture measures such as energy, contrast, entropy, correlation, dissimilarity, and homogeneity. These features were computed for each direction and their average was used.

#### Biophysical growth model-based features

These features were derived from tumor biophysical growth model that is part of GLISTR^33,34^. This model calculates diffusion information and mass-effect of the tumor and provides diffusion time, diffusion coefficient of white matter, and number of tumor foci.

A total of 267 features were extracted, including 24 texture features, 11 volumetric and shape features, 66 intensity features, 163 histogram features, 9 location features, and 3 biophysical growth model-based features (see Table S1 for more detail on features). All features were extracted with an in-house feature analysis program implemented in Matlab 2014b (Mathworks, Natick, Mass).

### Clustering

The K-means clustering algorithm was used to determine the underlying imaging subtypes of glioblastoma based on extracted features of the discovery cohort. Features were scaled [0–1] prior to the clustering process. Varying K from 2 to 6, we ran K-means 1000 times on the image features using the Euclidean distance metric and initializing random starting seeds in each iteration, and selected K that yielded the most stable and reproducible cluster assignments across permuted cluster runs calculated via an average adjusted rand index^37^. Later, within the 1000 iterations of selected K, the clustering assignment leading to highest average silhouette index (highest separability amongst the clusters) and appearing at-least 20% of all the iterations was chosen. This established the optimal number of intrinsic unsupervised clusters as defined by image features in the discovery cohort.

### Statistical analysis

The statistical analysis was performed with R software version 3.3.2 (http://www.R-project.org), SPSS (IBM), and Matlab, where appropriate.

For evaluation of statistically significant imaging features associated with each cluster, we used Kruskal-Wallis test^38^, and adjusted for multiple comparisons using Bonferroni corrections^39^. In addition, to assess the predictive performance of each feature to discriminate tumors with and without *EGFRvIII* within the discovered subtypes, the univariate analysis was performed using effect-size methodology^40^.

The association of the discovered subtypes with survival was assessed on the discovery cohort and validated on the replication cohort by using Kaplan-Meier survival analysis. The survival curves of the subtypes were compared statistically using a Cox proportional hazards model to evaluate statistical significance at 95% confidence interval. When comparing across-cluster molecular feature, we performed the Pearson χ2 test. The differences in age, sex and KPS between the discovery and the replication data sets were also assessed using Pearson χ2 test.

Further, we used Cox proportional hazards model to perform a time-to-event analysis by using several individual clinical features (age and location) and by augmenting subtype with these features. We predicted survival by estimating c-index^41^, a generalization of the area under the receiver operating characteristic curve.

### Spatial distribution calculation

The label (segmentation) maps of all the patients were spatially co-registered to a standardized atlas, using sophisticated deformable registration methods that accounted for both mass effect and inter-individual anatomical variations^33^. The spatial distribution probability for a particular subtype at voxel *i* was computed as the number of tumors of the subtype that intersect voxel *i* divided by the total number of tumors belonging to that particular subtype. A tumor was defined here as the cluster of voxels corresponding to enhancing and nonenhancing components^22^. In addition, the proportion of tumor was calculated in the regions defined by the standardized atlas. Overall brain was divided into nine anatomical regions (frontal,temporal, parietal, basal ganglia, insula, cc fornix, occipital, cerebellum, and brain stem) according to the atlas and the percentage of tumor (enhancing tumor + non-enhancing tumor core) for each region was calculated^22^.

### Within- vs. across-subtypes radiogenomic signatures of *EGFRvIII*-mutated tumors

We hypothesized that radiogenomic signatures would be more evident within individual subtypes rather than across all subtypes pooled together, since the clustering process results in a more homogenized set of sub-populations. We used *EGFRvIII* mutation as testbed for our hypothesis, since it is of therapeutic interest and is present in approximately 33% of glioblastomas^42,43^. The aberrations in its expression lead to reduced response to aggressive therapy, and poor survival^14^. To test the hypothesis, within-clusters SVM-based classifiers^44^ were built for the prediction of tumors with and without *EGFRvIII*, and were compared with a single universal SVM classifier built across clusters. The classifiers were built on the imaging features (used in clustering) of patients having *EGFRvIII* status available, which was a subset of all patients (see Table S2). In particular, radial basis function kernel of SVM was used for within-cluster classification. Features were selected on the training data by using SVM sequential feature selection in each iteration of cross-validation and the selected features were used to identify tumors with and without *EGFRvIII*.

### Validation of the reproducibility of subtypes

The reproducibility of the subtypes was validated via 10-fold cross-validation in the discovery cohort. In each iteration, 9 folds were used for establishing the subtypes, and the clustering assignments of the subjects of the 10^*th*^ fold were calculated based on the proximity of their features to the means of the established clusters.

The reproducibility of the discovered subtypes was further validated on an independent replication cohort based on the means of the discovery cohort. The features in the replication cohort were normalized based on the scaling performed on the discovery cohort. Later, the Euclidean distance between the features of each subject of replication cohort and the center (mean) of each cluster was calculated, and the subject was assigned to the cluster with minimum Euclidean distance. The survival rates, *IDH1* mutational status, and *MGMT* methylation status of the subjects of replication cohort were analyzed.

### Analysis of the complementary information provided by subtypes on survival prediction

Certain clinical, demographic and imaging biomarkers such as age and anatomical location of the tumor in brain have diagnostic value. We hypothesized that those clinical and imaging biomarkers when combined with the proposed imaging subtypes yield better prediction of survival. We used Cox proportional hazard model in six configurations to predict survival using: i) age, ii) location, iii) subtype, iv) subtype and age, v) subtype and location, and vi) age, location and subtype, altogether.

## Electronic supplementary material

supplementary material
