# Supplementary material for: Radiomic MRI signature reveals three distinct subtypes of glioblastoma with different clinical and molecular characteristics, offering prognostic value beyond IDH1
Source: Sci Rep. 2018 Mar 23;8:5087. doi: 10.1038/s41598-018-22739-2 (PMC5865162; doi:10.1038/s41598-018-22739-2)
Supplement: Supplementary file 1 — supplementary material [file 41598_2018_22739_MOESM1_ESM.docx]

**Radiomic MRI signature reveals three distinct subtypes of glioblastoma with different clinical and molecular characteristics, offering prognostic value beyond *IDH1***

**Saima Rathore^1,2^**^†^**, Hamed Akbari^1,2^**^†^**, Martin Rozycki^1,2^, Kalil G. Abdullah****^3^,** **MacLean P. Nasrallah^4^, Zev A. Binder^3^, Ramana V. Davuluri^5^,** **Robert A. Lustig^6^, Nadia Dahmane****^3^, Michel Bilello^1,2^, Donald M.** **O’Rourke^3^, Christos Davatzikos^1,2*^**

^1^Department of Radiology, Perelman School of Medicine; ^2^Center for Biomedical Image Computing and Analytics, Perelman School of Medicine; ^3^Department of Neurosurgery, Perelman School of Medicine^; 4^Department of Pathology and Laboratory Medicine, Perelman School of Medicine, University of Pennsylvania, Philadelphia, PA, USA. ^5^Department of Biomedical Informatics, Northwestern University Feinberg School of Medicine, Chicago, IL, USA. ^6^Department of Radiation Oncology, Perelman School of Medicine, University of Pennsylvania, Philadelphia, PA, USA.

**S1. Data acquisition protocol**

Pre-operative magnetic resonance images (MRIs) were acquired using a 3 Tesla scanner. Obtained for all patients prior to surgery were: T1-weighted (T1): matrix 192 × 256 × 192; resolution 0.98 × 0.98 × 1.00 mm^3^; repetition time (TR): 1760 ms; echo time (TE): 3.1 ms; T1 with contrast-enhanced (T1CE): matrix 192 × 256 × 192; resolution 0.98 × 0.98 × 1.00; TR: 1760 ms; TE: 3.1 ms. T2-weighted (T2): matrix 210 × 256 × 64; resolution 0.94 × 0.94 × 3.00; TR: 4680 ms; TE: 85 ms. T2 fluid-attenuated inversion recovery (FLAIR): matrix 192 × 256 × 60; resolution 0.94 × 0.94 × 3.00; TR: 9420 ms; TE: 141 ms. Diffusion tensor imaging (DTI): matrix 128 × 128 × 40; resolution 1.72 × 1.72 × 3.00; 30 gradient directions. Dynamic susceptibility contrast-enhanced (DSC)-MRI, gradient echo type echo planar imaging (GRE EPI) = field of view (FOV) 22 cm 128 × 128 × 20; resolution 1.72 × 1.72 × 3 mm^3^; TR: 2000 ms; TE: 45 ms. An initial loading dose of one-quarter of the total contrast dose was administered first to help minimize errors due to potential contrast leakage out of intravascular space, and DSC-MRI data were acquired during a second bolus of the remaining contrast dose after a 5-min delay for a total of 0.3 mL/kg or 1.5 times single dose MultiHance (gadobenate dimeglumine). For post-processing, blood volume maps were created on a Leonardo workstation (Siemens) using the Neuro Perfusion Evaluation task card as per clinical routine.

**S2. Inclusion criteria and study source**

Inclusion of patients was based on the following criteria: (i) age over 18 years, (ii) histo-pathological tissue diagnosis of glioblastoma (WHO grade IV), (iii) availability of 3 Tesla scanner data, and (iv) available pre-operative MRI consisting of pre-contrast axial T1, T1CE, T2, FLAIR, DTI, and DSC-MRI. The inclusion criterion for survival analysis was gross total resection of tumor. For molecular subtypes and epidermal growth factor receptor variant –III (*EGFRvIII*) analyses, the inclusion criterion was availability of data. Molecular subtypes and *EGFRvIII* mutation status were determined by the methods described in Pal et al.[^1^](#_ENREF_1) and Section S3, respectively. All patients were treated according to standard of care, which included maximal safe resection, radiotherapy to 6000 cGy, and concomitant and adjuvant chemotherapy with temozolomide^[2](#_ENREF_2)^. The [Karnofsky performance status (KPS) of the patients was obtained from the clinical records.](#_ENREF_34) The study was approved by the institutional review board of the University of Pennsylvania. The isocitrate dehydrogenase-1 (*IDH1*) mutation status and O^6^-methylguanine–DNA methyltransferase methylation status for the replication cohort were acquired from the clinical records. The *IDH1* mutation status was determined by next generation sequencing.

**S3. Determination of *EGFRvIII* mutation status**

The most representative block per resected tissue specimen was chosen by a board certified neuropathologist on the basis of morphology for further genetic analysis. Total nucleic acids, enriched for RNA was extracted from the submitted Formalin-fixed Paraffin-embedded specimen according to manufacturer`s instructions (BeckmanCoulter, Inc., [Brea, California](https://en.wikipedia.org/wiki/Brea,_California).). cDNA was then synthesized from RNA, allowing detection of genomic rearrangements in an "intron-free environment" and allowing direct detection of *EGFR* normal and variant gene expression. Polymerase chain reaction (PCR) primers were designed to capture exons 1-2 (wildtype) and exons 1 to 8 (*EGFRvIII* mutant). In addition, three primer sets within *EGFR* are included to detect the level of RNA degradation in the sample using these sets with increasing target sizes (9-9: 93bp, 9-10: 141bp and 9-12: 251bp) and internal housekeeping genes expressed in the brain.

Sequencing of PCR-enriched libraries was performed on the Illumina MiSeq platform using multiplexed, paired-end reads.  Analysis and interpretation utilized a customized bioinformatics process. All variants listed are with reference to the hg19 Genome build.

This assay detects the presence or absence of the *EGFRvIII* directly demonstrating the presence of the critical “glycine” at the exon1-8 boundary and determines the relative burden of *EGFRvIII* to wildtype *EGFR*. This assay detects variants representing at least 5% of the total sequence reads. A bioinformatics pipeline, ‘*EGFRvIII* Picker v1.1’, was used to process the data and identify normal and abnormal reads.

**Tables**

Table S1: Description of the features used for unsupervised clustering. ED =Edema, NC = Non-enhancing tumor core, TU = Enhancing tumor core, PH = Peak height, PSR = Percent signal recovery, RCBV = Relative cerebral blood volume, TR = Diffusion trace signal, FA = Fractional anisotropy, RAD = Radial diffusivity, AX = Axial diffusivity.

| **Feature name** | **Feature description** |
| --- | --- |
| N_ED_VOL | Normalized volume of edema w.r.t total brain size |
| N_NC_VOL | Normalized volume of non-enhancing tumor core w.r.t total brain size |
| N_TU_VOL | Normalized volume of enhancing tumor |
| N_TU_VOL/  N_NC_VOL | Ratio of volume of enhancing tumor to volume of non-enhancing tumor  core |
| N_ED_VOL  /(N_NC_VOL+  N_ED_VOL+  N_TU_VOL) | Ratio of volume of edema to the volume of whole tumor (combined volume of edema, non-enhancing tumor core and enhancing tumor) |
| No. of Tumors | Number of tumors in the brain |
| DW | Diffusion of tumor in white matter (a parameter measured by biophysical growth model) |
| Time | Estimated growth time by the biophysical tumor growth model |
| ED_MEAN_T1 | Average T1 signal in edema |
| ED_MEAN_T1CE | Average T1CE signal in edema |
| ED_MEAN_T2 | Average T2 signal in edema |
| ED_MEAN_FLAIR | Average FLAIR signal in edema |
| ED_MEAN_AX | Average axial diffusivity in edema |
| ED_MEAN_FA | Average fractional anisotropy in edema |
| ED_MEAN_RAD | Average radial diffusivity in edema |
| ED_MEAN_TR | Average trace in edema |
| ED_MEAN_PH | Average peak height in edema |
| ED_MEAN_RCBV | Average relative cerebral blood volume in edema |
| ED_MEAN_PSR | Average percent signal recovery in edema |
| NC_MEAN_T1 | Average T1 signal in non-enhancing tumor core |
| NC_MEAN_T1CE | Average T1CE signal in non-enhancing tumor core |
| NC_MEAN_T2 | Average T2 signal in non-enhancing tumor core |
| NC_MEAN_FLAIR | Average FLAIR signal in non-enhancing tumor core |
| NC_MEAN_AX | Average axial diffusivity in non-enhancing tumor core |
| NC_MEAN_FA | Average fractional anisotropy in non-enhancing tumor core |
| NC_MEAN_RAD | Average radial diffusivity in non-enhancing tumor core |
| NC_MEAN_TR | Average trace in non-enhancing tumor core |
| NC_MEAN_PH | Average peak height in non-enhancing tumor core |
| NC_MEAN_RCBV | Average relative cerebral blood volume in non-enhancing tumor core |
| NC_MEAN_PSR | Average percent signal recovery in non-enhancing tumor core |
| TU_MEAN_T1 | Average T1 signal in enhancing tumor |
| TU_MEAN_T1CE | Average T1CE signal in enhancing tumor |
| TU_MEAN_T2 | Average T2 signal in enhancing tumor |
| TU_MEAN_FLAIR | Average FLAIR signal in enhancing tumor |
| TU_MEAN_AX | Average axial diffusivity in enhancing tumor |
| TU_MEAN_FA | Average fractional anisotropy in enhancing tumor |
| TU_MEAN_RAD | Average radial diffusivity in enhancing tumor |
| TU_MEAN_TR | Average trace in enhancing tumor |
| TU_MEAN_PH | Average peak height in enhancing tumor |
| TU_MEAN_RCBV | Average relative cerebral blood volume in enhancing tumor |
| TU_MEAN_PSR | Average percent signal recovery in enhancing tumor |
| ED_STD_T1 | Std. deviation of T1 signal in edema |
| ED_STD_T1CE | Std. deviation of T1CE signal in edema |
| ED_STD_T2 | Std. deviation of T2 signal in edema |
| ED_STD_FLAIR | Std. deviation of FLAIR signal in edema |
| ED_STD_AX | Std. deviation of axial diffusivity in edema |
| ED_STD_FA | Std. deviation of fractional anisotropy in edema |
| ED_STD_RAD | Std. deviation of radial diffusivity in edema |
| ED_STD_TR | Std. deviation of trace in edema |
| ED_STD_PH | Std. deviation of peak height in edema |
| ED_STD_RCBV | Std. deviation of relative cerebral blood volume in edema |
| ED_STD_PSR | Std. deviation of percent signal recovery in edema |
| NC_STD_T1 | Std. deviation of T1 signal in non-enhancing tumor core |
| NC_STD_T1CE | Std. deviation of T1CE signal in non-enhancing tumor core |
| NC_STD_T2 | Std. deviation of T2 signal in non-enhancing tumor core |
| NC_STD_FLAIR | Std. deviation of FLAIR signal in non-enhancing tumor core |
| NC_STD_AX | Std. deviation of axial diffusivity in non-enhancing tumor core |
| NC_STD_FA | Std. deviation of fractional anisotropy in non-enhancing tumor core |
| NC_STD_RAD | Std. deviation of radial diffusivity in non-enhancing tumor core |
| NC_STD_TR | Std. deviation of trace in non-enhancing tumor core |
| NC_STD_PH | Std. deviation of peak height in non-enhancing tumor core |
| NC_STD_RCBV | Std. deviation of relative cerebral blood volume in non-enhancing tumor  core |
| NC_STD_PSR | Std. deviation of percent signal recovery in non-enhancing tumor core |
| TU_STD_T1 | Std. deviation of T1 signal in enhancing tumor |
| TU_STD_T1CE | Std. deviation of T1CE signal in enhancing tumor |
| TU_STD_T2 | Std. deviation of T2 signal in enhancing tumor |
| TU_STD_FLAIR | Std. deviation of FLAIR signal in enhancing tumor |
| TU_STD_AX | Std. deviation of axial diffusivity in enhancing tumor |
| TU_STD_FA | Std. deviation of fractional anisotropy in enhancing tumor |
| TU_STD_RAD | Std. deviation of radial diffusivity in enhancing tumor |
| TU_STD_TR | Std. deviation of trace in enhancing tumor |
| TU_STD_PH | Std. deviation of peak height in enhancing tumor |
| TU_STD_RCBV | Std. deviation of relative cerebral blood volume in enhancing tumor |
| TU_STD_PSR | Std. deviation of percent signal recovery in enhancing tumor |
| ED_BINS_T1_1-5 | Percentage of edema voxels in Bin #1-5 of T1 |
| NC_BINS_T1_1-5 | Percentage of non-enhancing tumor core voxels in Bin #1-5 of T1 |
| TU_BINS_T1_1-5 | Percentage of enhancing tumor voxels in Bin #1-5 of T1 |
| ED_BINS_T1CE_1-5 | Percentage of edema voxels in Bin #1-5 of T1CE |
| NC_BINS_T1CE_1-5 | Percentage of non-enhancing tumor core voxels in Bin #1-5 of T1CE |
| TU_BINS_T1CE_1-5 | Percentage of enhancing tumor voxels in Bin #1-5 of T1CE |
| ED_BINS_T2_1-5 | Percentage of edema voxels in Bin #1-5 of T2 |
| NC_BINS_T2_1-5 | Percentage of non-enhancing tumor core voxels in Bin #1-5 of T2 |
| TU_BINS_T2_1-5 | Percentage of enhancing tumor voxels in Bin #1-5 of T2 |
| ED_BINS_FLAIR_1-5 | Percentage of edema voxels in Bin #1-5 of FLAIR |
| NC_BINS_FLAIR_1-5 | Percentage of non-enhancing tumor core voxels in Bin #1-5 of FLAIR |
| TU_BINS_FLAIR_1-5 | Percentage of enhancing tumor voxels in Bin #1-5 of FLAIR |
| ED_BINS_AX_1-5 | Percentage of edema voxels in Bin #1-5 of axial diffusivity |
| NC_BINS_AX_1-5 | Percentage of non-enhancing tumor core voxels in Bin #1-5 of axial  diffusivity |
| TU_BINS_AX_1-5 | Percentage of enhancing tumor voxels in Bin #1-5 of axial diffusivity |
| ED_BINS_FA_1-5 | Percentage of edema voxels in Bin #1-5 of fractional anisotropy |
| NC_BINS_FA_1-5 | Percentage of non-enhancing tumor core voxels in Bin #1-5 of fractional anisotropy |
| TU_BINS_FA_1-5 | Percentage of enhancing tumor voxels in Bin #1-5 of fractional anisotropy |
| ED_BINS_RAD_1-5 | Percentage of edema voxels in Bin #1-5 of radial diffusivity |
| NC_BINS_RAD_1-5 | Percentage of non-enhancing tumor core voxels in Bin #1-5 of radial diffusivity |
| TU_BINS_RAD_1-5 | Percentage of enhancing tumor voxels in Bin #1-5 of radial diffusivity |
| ED_BINS_TR_1-5 | Percentage of edema voxels in Bin #1-5 of trace |
| NC_BINS_TR_1-5 | Percentage of non-enhancing tumor core voxels in Bin #1-5 of trace |
| TU_BINS_TR_1-5 | Percentage of enhancing tumor voxels in Bin #1-5 of trace |
| ED_BINS_PH_1-5 | Percentage of edema voxels in Bin #1-5 of peak height |
| NC_BINS_PH_1-5 | Percentage of non-enhancing tumor core voxels in Bin #1-5 of peak height |
| TU_BINS_PH_1-5 | Percentage of enhancing tumor voxels in Bin #1-5 of peak height |
| ED_BINS_RCBV_1-5 | Percentage of edema voxels in Bin #1-5 of relative cerebral blood volume |
| NC_BINS_RCBV_1-5 | Percentage of non-enhancing tumor core voxels in Bin #1-5 of relative  cerebral blood volume |
| TU_BINS_RCBV_1-5 | Percentage of enhancing tumor voxels in Bin #1-5 of relative cerebral blood volume |
| ED_BINS_PSR_1-5 | Percentage of edema voxels in Bin #1-5 of percent signal recovery |
| NC_BINS_PSR_1-5 | Percentage of non-enhancing tumor core voxels in Bin #1-5 of percent signal recovery |
| TU_BINS_PSR_1-5 | Percentage of enhancing tumor voxels in Bin #1-5 of percent signal recovery |
| ED_T1CE_Energy | Texture measure of Energy for edema in T1CE |
| ED_ T1CE _Contrast | Texture measure of Contrast for edema in T1CE |
| ED_ T1CE _Entropy | Texture measure of Entropy for edema in T1CE |
| ED_ T1CE _Homogeneity | Texture measure of Homogeneity for edema in T1CE |
| ED_ T1CE _Correlation | Texture measure of Correlation for edema in T1CE |
| ED_ T1CE _SumAverage | Texture measure of SumAverage for edema in T1CE |
| ED_ T1CE _Variance | Texture measure of Variance for edema in T1CE |
| ED_ T1CE _Dissimilarity | Texture measure of Dissimilarity for edema in T1CE |
| NC_ T1CE _Energy | Texture measure of Energy for non-enhancing tumor core in T1CE |
| NC_ T1CE _Contrast | Texture measure of Contrast for non-enhancing tumor core in T1CE |
| NC_ T1CE _Entropy | Texture measure of Entropy for non-enhancing tumor core in T1CE |
| NC_ T1CE _Homogeneity | Texture measure of Homogeneity for non-enhancing tumor core in T1CE |
| NC_ T1CE _Correlation | Texture measure of Correlation for non-enhancing tumor core in T1CE |
| NC_ T1CE _SumAverage | Texture measure of SumAverage for non-enhancing tumor core in T1CE |
| NC_ T1CE _Variance | Texture measure of Variance for non-enhancing tumor core in T1CE |
| NC_ T1CE _Dissimilarity | Texture measure of Dissimilarity for non-enhancing tumor core in T1CE |
| TU_ T1CE _Energy | Texture measure of Energy for enhancing tumor in T1CE |
| TU_ T1CE _Contrast | Texture measure of Contrast for enhancing tumor in T1CE |
| TU_ T1CE _Entropy | Texture measure of Entropy for enhancing tumor in T1CE |
| TU_ T1CE _Homogeneity | Texture measure of Homogeneity for enhancing tumor in T1CE |
| TU_T1CE_Correlation | Texture measure of Correlation for enhancing tumor in T1CE |
| TU_ T1CE _SumAverage | Texture measure of SumAverage for enhancing tumor in T1CE |
| TU_ T1CE _Variance | Texture measure of Variance for enhancing tumor in T1CE |
| TU_ T1CE _Dissimilarity | Texture measure of Dissimilarity for enhancing tumor in T1CE |
| NC_SPH | Sphericity of non-enhancing tumor core |
| NC_CIR | Circularity of non-enhancing tumor core |
| TUM_SPH | Sphericity of tumor core |
| TUM_CIR | Circularity of tumor core |
| WTUM_SPH | Sphericity of whole tumor |
| WTUM_CIR | Circularity of whole tumor |

Table S2: Demographics of the discovery cohort of glioblastoma subjects (N = 208). Percentages for *EGFRvIII* mutation and molecular subtypes are in terms of the available number of subjects.

| **Characteristics** | **Category** | **Value** |
| --- | --- | --- |
| **Age (Years)** | | |
|  | MEAN | 62.30 |
|  | Std. deviation | 12.63 |
| **Gender** |  |  |
|  | Male, *n* (%) | 120 (57.69) |
|  | Female, *n* (%) | 88 (42.31) |
| **Survival** |  |  |
|  | Available after excluding residual, *n* (%) | 151 (72.59) |
| ***EGFRvIII*** | | |
|  | Available, *n* (%) | 107 (51.44) |
|  | Tumors with *EGFRvIII*, *n* (%) | 33 (30.84) |
|  | Tumors without *EGFRvIII*, *n* (%) | 74 (69.15) |
| **Molecular subtype** | | |
|  | Available, *n* (%) | 78 (37.50) |
|  | Proneural, *n* (%) | 13 (16.67) |
|  | Neural, *n* (%) | 22 (28.21) |
|  | Mesenchymal, *n* (%) | 23 (29.49) |
|  | Classical, *n* (%) | 20 (25.64) |

Table S3: Demographics of the replication cohort of glioblastoma subjects (N = 53).

| **Characteristics** | **Category** | **Value** |
| --- | --- | --- |
| **Age (Years)** | | |
|  | MEAN | 61.96 |
|  | Std. deviation | 9.48 |
| **Gender** |  |  |
|  | Male, *n* (%) | 31 (58.49) |
|  | Female, *n* (%) | 22 (41.51) |
| **Survival** |  |  |
|  | Available after excluding residual, *n* (%) | 46 (86.79) |

Table S4: Karnofsky Performance Status of the subjects in complete discovery cohort and within different subtypes.

|  | **Complete dataset** | **Rim-enhancing** | **Irregular** | **Solid** |
| --- | --- | --- | --- | --- |
| MEAN | 74.44 | 73.91 | 75.09 | 74.11 |
| Std. deviation | 11.46 | 14.69 | 8.69 | 12.34 |
| Median | 70.00 | 70.00 | 80.00 | 70.00 |
| Range | 30-100 | 30-100 | 60-90 | 50-100 |

**Figures**

**
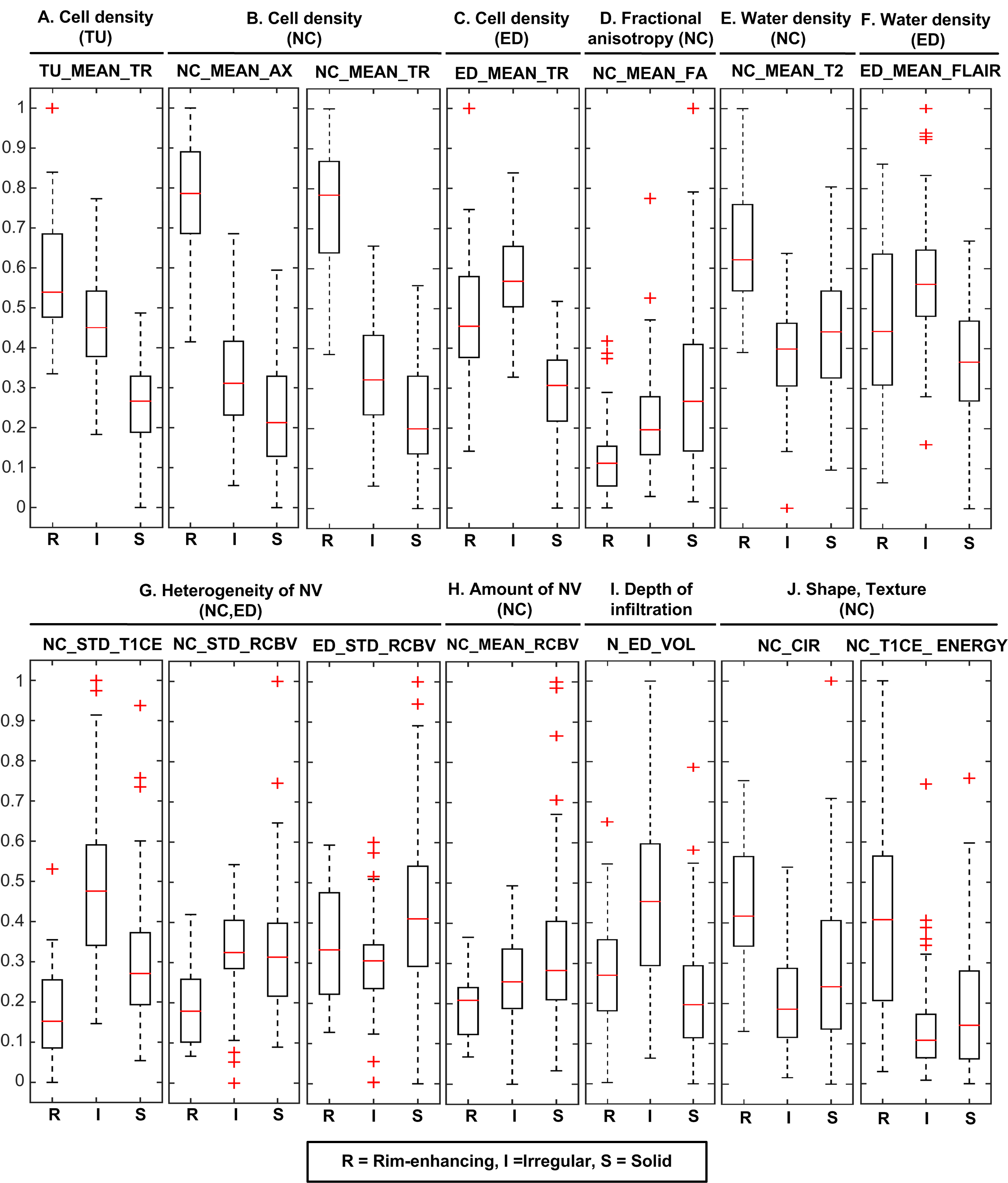
**

**Fig S1: Image features significantly associated with each subtype calculated via ANOVA and effect size methods.** This diagram represents different features shown in Figure 2 of the main manuscript. Instead of the histogram binning based features, average values are shown here for some features for clarity. Results are shown only for the discovery cohort. P<0.01 for all the features.


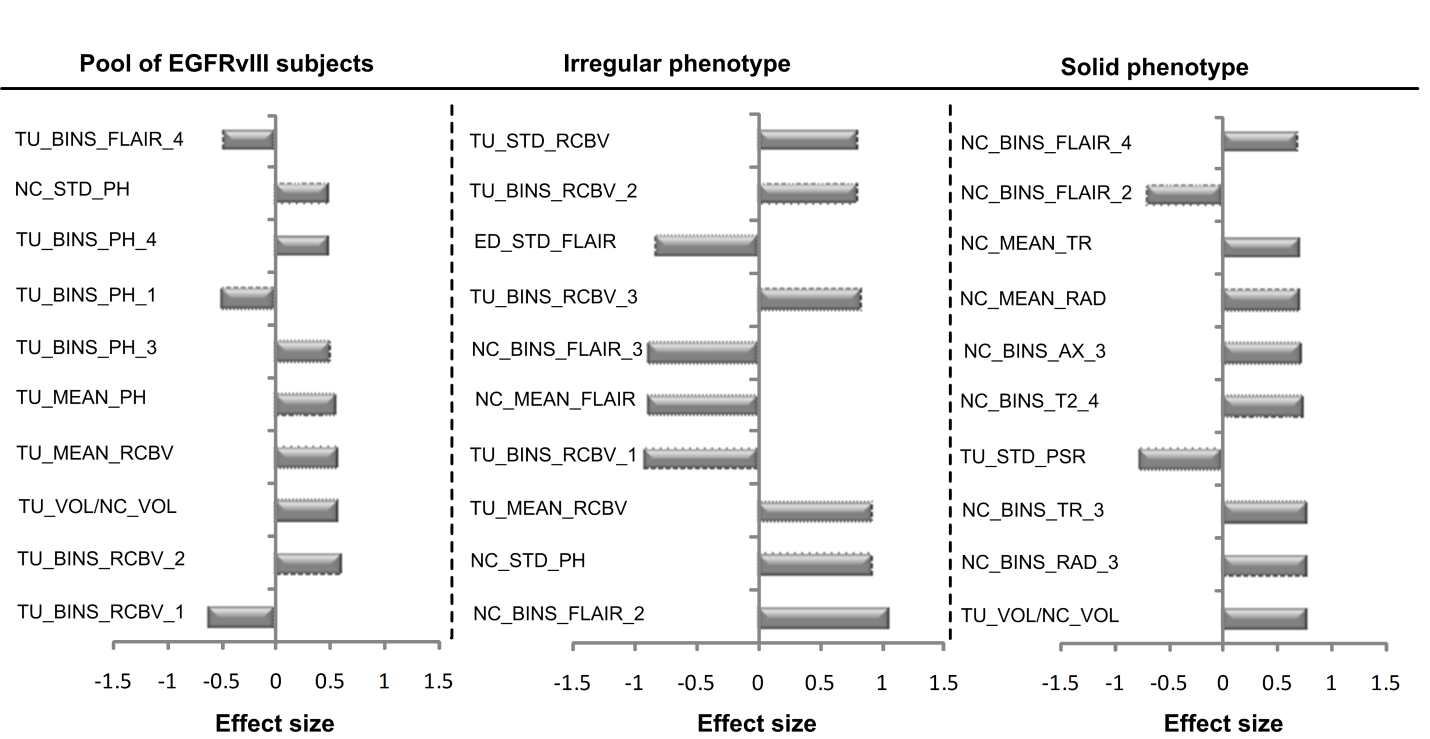


**Pool of *EGFRvIII* subjects**

**Solid subtype**

**Irregular subtype**

**Fig S2: Effect sizes of the top-most 10 discriminative features between tumors with and without *EGFRvIII* from the pool of tumors with *EGFRvIII* (left), and individually in each of the two subtypes that had included the majority of *EGFRvIII*-mutated tumors.** Features are in decreasing discriminative power from bottom-to-top. Positive and negative values of effect size, respectively, show higher and lower value of that feature for *EGFRvIII*-mutated tumors.

**
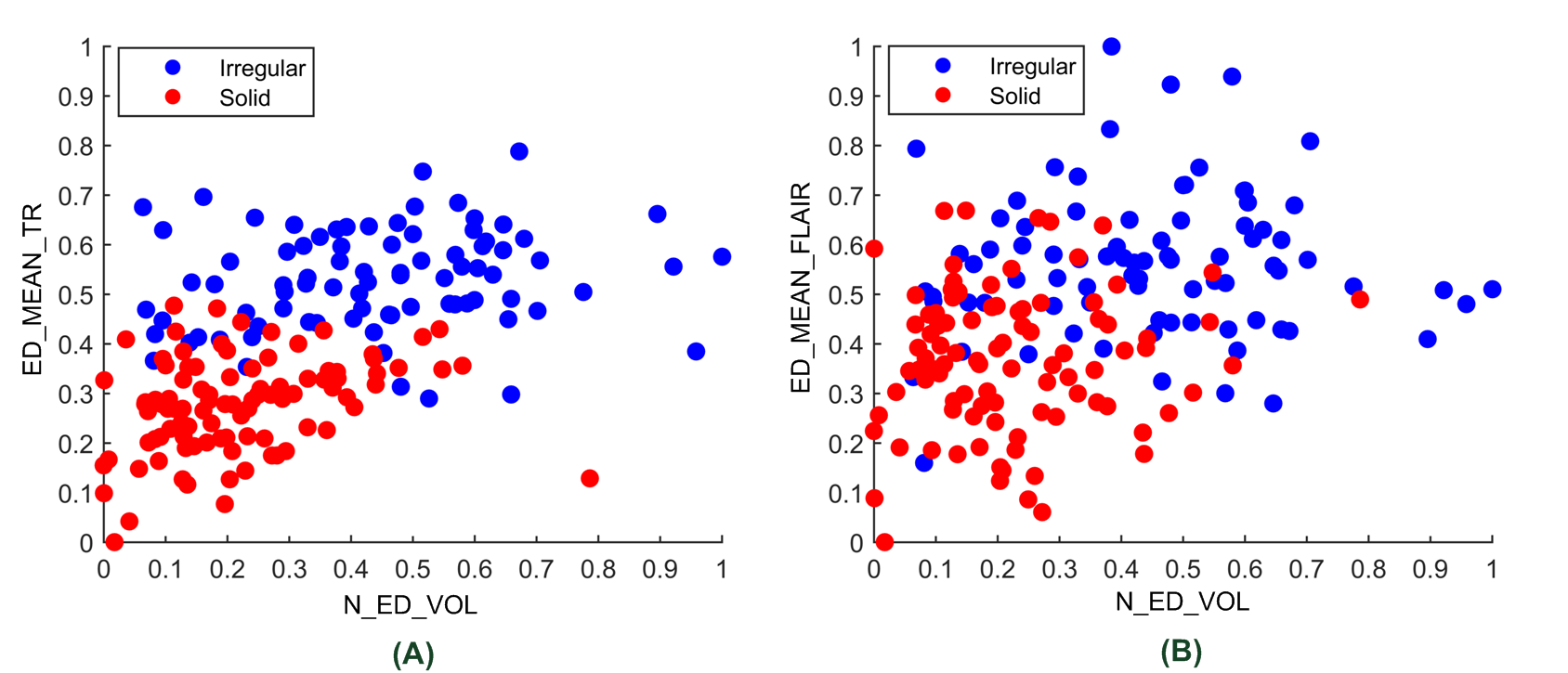
**

**Fig S3. Heterogeneity in the peritumoral edema of irregular and solid subtypes.** **A.** x-axis shows normalized volume of edema and y-axis shows average trace in edema. **B.** x-axis shows normalized volume of edema and y-axis shows average FLAIR signal in edema.

**
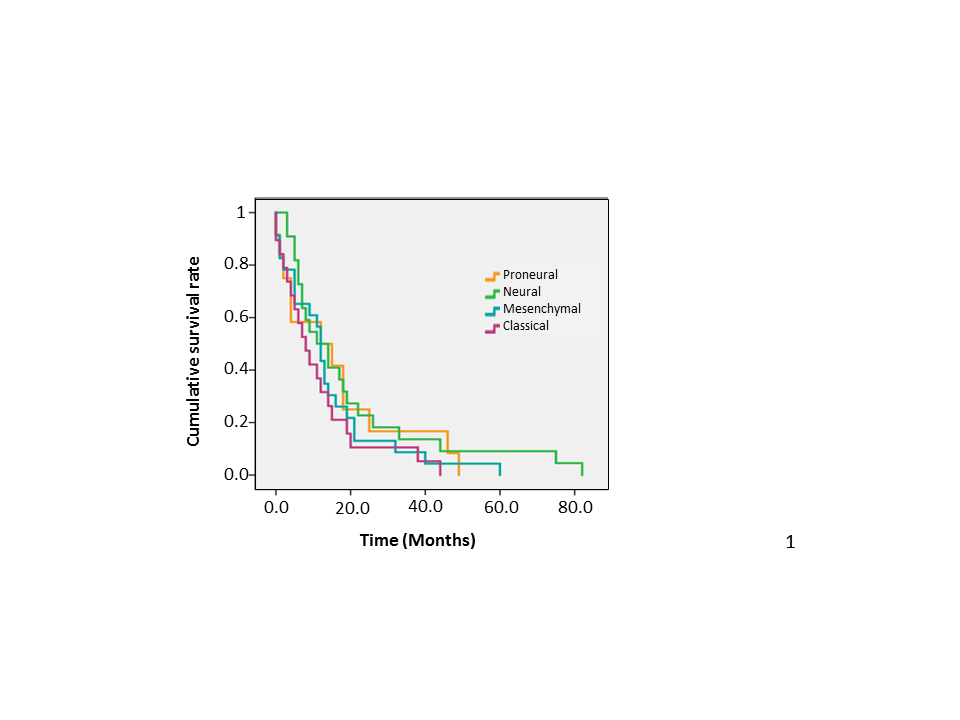
**

**Fig S4. Kaplan–Meier survival curves of the molecular subtypes.** Actual survival on x-axis is compared among each of the four molecular subtypes (*p*=0.08, log-rank test; hazard ratio = 1.01, 95% CI: 0.90-1.14, Cox proportional hazard model).

**
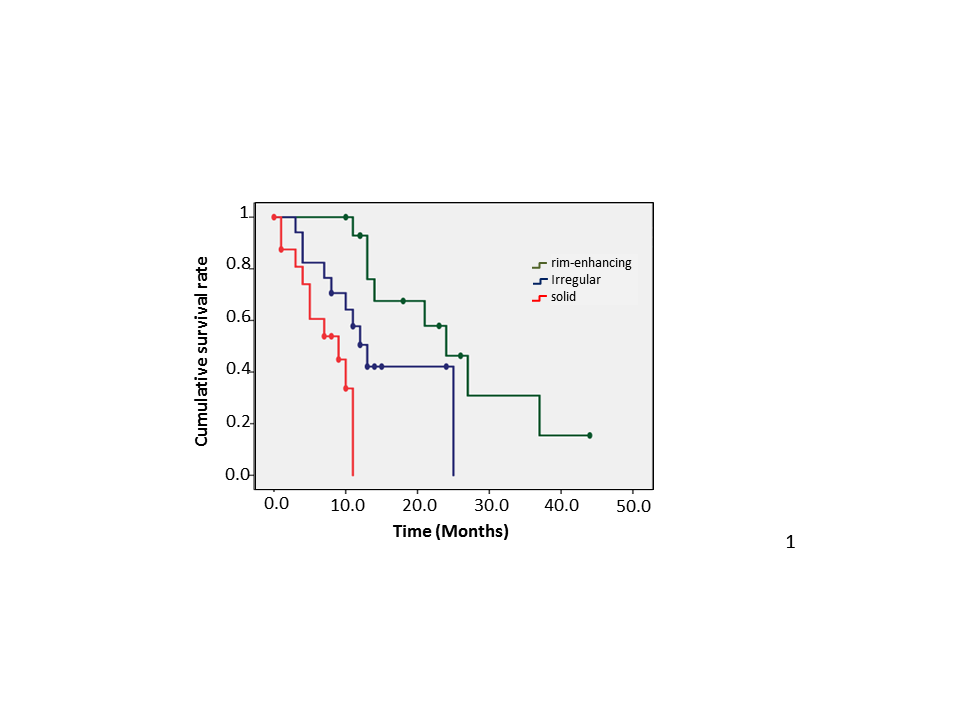
**

**Fig S5. Kaplan–Meier survival curves of the imaging subtypes within replication cohort.** Actual survival on x-axis is compared among each of the three imaging subtypes (*p*<0.001, log-rank test; hazard ratio = 3.74, 95% CI: 3.01-4.65, Cox proportional hazard model).


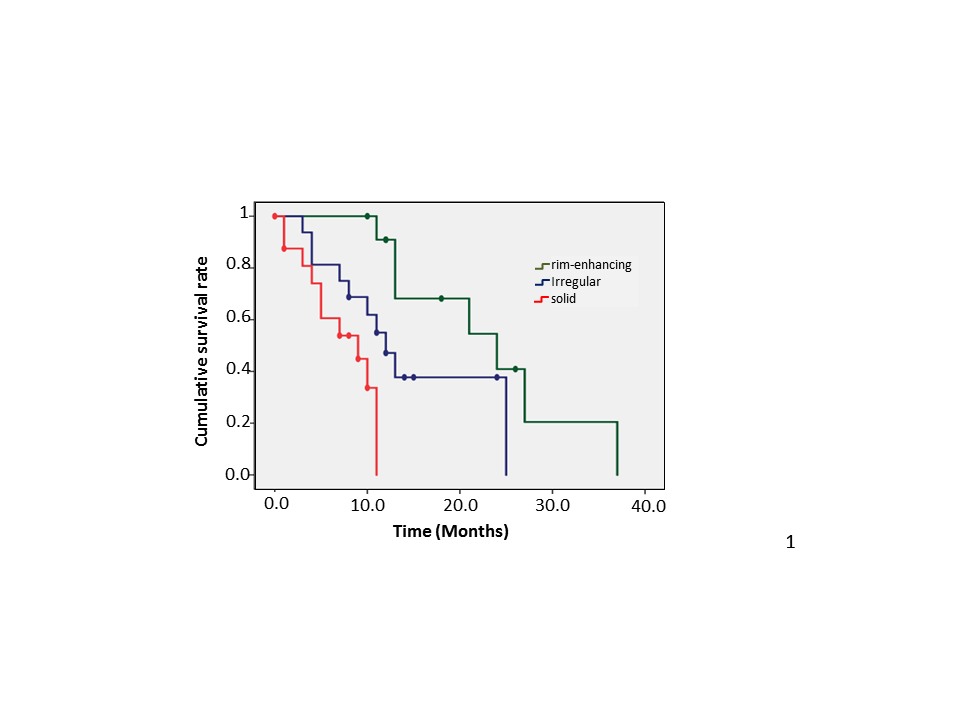


**Fig S6. Kaplan–Meier survival curves of the imaging subtypes within *IDH1*-wildtype patients.** Actual survival on x-axis is compared among each of the three imaging subtypes (*p*<0.001, log-rank test; hazard ratio = 3.38, 95% CI: 2.64- 4.34, Cox proportional hazard model).

**References**

1. Pal S, Bi Y, Macyszyn L, Showe LC, O'Rourke DM, Davuluri RV. Isoform-level gene signature improves prognostic stratification and accurately classifies glioblastoma subtypes. *Nucleic Acids Research.* 2014; 42(8):e64.

2. Stupp R, Hegi ME, Mason WP, et al. Effects of radiotherapy with concomitant and adjuvant temozolomide versus radiotherapy alone on survival in glioblastoma in a randomised phase III study: 5-year analysis of the EORTC-NCIC trial. *The Lancet Oncology.* 2009; 10(8):459-466.
